# Is There a Difference in the Prevalence of Gastric Ulcers between Stallions Used for Breeding and Those Not Used for Breeding?

**DOI:** 10.3390/ani14111531

**Published:** 2024-05-22

**Authors:** Sara Busechian, Francesca Bindi, Camillo Pieramati, Simona Orvieto, Lorenzo Pisello, Selene Cozzi, Flaminia Ortolani, Fabrizio Rueca

**Affiliations:** 1 Department of Veterinary Medicine, University of Perugia, Via San Costanzo 4, 06126 Perugia, Italy; camillo.pieramati@unipg.it (C.P.); ortolani.flaminia@gmail.com (F.O.); fabrizio.rueca@unipg.it (F.R.); 2 Department of Veterinary Sciences, University of Pisa, Via delle Piagge, 56124 Pisa, Italy; francesca.bindi@phd.unipi.it; 3 Independent Researcher, 06100 Perugia, Italy; simona.orvieto@gmail.com (S.O.); pisello.lorenzo@gmail.com (L.P.); 4 Independent Researcher, 20100 Milan, Italy; selene.c96@gmail.com

**Keywords:** Equine Squamous Gastric Disease, Equine Glandular Gastric Disease, breeding stallions, intact males

## Abstract

**Simple Summary:**

Equine gastric ulcer syndrome is a common disease in horses worldwide, with different prevalences in various categories and breeds. It is divided into two different illnesses, based on the mucosa affected: Equine Squamous Gastric Disease, if it involves the squamous mucosa, and Equine Glandular Gastric Disease, if the lesions are located in the glandular portion of the stomach. The effect of sex on gastric ulcers, especially on the squamous mucosa, is not completely elucidated: some studies found a higher prevalence of lesions of the squamous mucosa in geldings and stallions, while others failed to identify a sex as a risk factor. The few studies on glandular lesions did not show any correlation between sex and ulcers. A paper investigating only Thoroughbred mares at pasture found that about 70% of them had gastric ulcers, especially on the squamous mucosa. The aim of this study was to determine the prevalence of the disease in a population of male horses, and determine if their breeding or sports career can influence the development of gastric lesions. Gastroscopies were performed in 101 males, after recording data about their breeding and exercise history, management, and presence of clinical signs. Breeding activity does not determine the presence or severity of gastric lesions in either mucosa, while exercise and a combination of both exercise and breeding is associated with ulcers in the glandular mucosa. Clinical signs are correlated only with the severity of lesions in the squamous one.

**Abstract:**

Equine gastric ulcer syndrome (EGUS) is a worldwide disease, and includes two different syndromes, Equine Squamous Gastric Disease (ESGD), affecting the squamous mucosa, and Equine Glandular Gastric Disease (EGGD), affecting the glandular mucosa. These two diseases are present in different categories (different activities, ages, etc.) and breeds of horses. The effect of sex on gastric health is not clear: some studies found a higher prevalence of ESGD in geldings and stallions, while others found no influence of sex on the squamous mucosa. The few studies conducted on glandular diseases failed to identify sex as a risk factor. The only study on breeding horses, focused on Thoroughbred mares at pasture, found that 70% of them were affected by gastric ulcerations especially in the squamous mucosa. The aim of this study was to determine the prevalence of EGUS, ESGD, and EGGD in intact males while also investigating the potential influences of breeding and exercise activity on the occurrence and severity of the diseases. A total of 101 intact males were admitted for gastroscopic examination. Comprehensive data regarding their breeding and sports history, management, and presence of clinical signs were recorded. A statistical analysis was performed. Within this equine population, no discernible relationship was found between breeding activity and occurrence and severity of ESGD or EGGD. Exercise and a combination of exercise and breeding activities were found to be associated with the occurrence of EGGD. The presence of clinical signs was correlated only with the grade of ESGD in this cohort of horses.

## 1. Introduction

Equine Gastric Ulcer Syndrome (EGUS) is a worldwide disease with a high prevalence across all categories of horses [1,2,3,4,5,6,7]. The ECEIM consensus statement divides the disease in two different illnesses, with different pathophysiology, localization of lesions, and treatment: Equine Squamous Gastric Disease (ESGD), on the squamous mucosa, and Equine Glandular Gastric Disease (EGGD), on the glandular mucosa [1,2,4,5,6,7]. Clinical signs are variable, from recurrent colic and weight loss, to reduced appetite, but most horses are asymptomatic [1,2,3,4,5,7,8].

Risk factors for EGUS are associated with increased stress levels, especially exercise, but also transportation and changes in management and herd dynamics [1,2,4,5,6,7,9]. Dietary factors also influence the development of ESGD, especially the amount of grain fed and the number of feedings per day [1,2,3,4,5,6]. The association between sex and gastric ulcers is still an open issue: considering ESGD, some studies report no effect, while others indicate a higher risk of gastric ulcers in geldings or stallions [2,5,10,11,12]. Investigations into EGGD are relatively limited, and in the few studies published, no sex-related effect was found [1,4,7,13,14].

The welfare of breeding stallions raised concerns, especially given their management practices. They are typically confined to individual stalls with minimal access to paddocks and limited social interactions, especially during the breeding season [15]. Furthermore, certain stallions may serve a dual role—breeding and sports—thus increasing their risk factors for the development of EGUS (exercise, traveling, high amounts of starch in their diet, etc. [1,2,3,4,5,6,7,13,14,16]). For non-breeding stallions, uniform management is lacking, especially in younger horses before the start of their breeding or sports career, where the owner’s preference and activity of the horse influence housing and feeding regimes. Both categories of stallions, though, are usually exposed to mares, both at home and during their sports and breeding careers, but the extent of interaction may differ between the different animals and management systems. Despite the absence of specific data on the prevalence and risk factors for gastric ulcers in breeding stallions, a study on Thoroughbred broodmares at pasture identified about 70% of animals affected [17]. This highlights the need for further studies on breeding animals, to identify the presence of specific risk factors of EGUS related to the reproductive career of horses.

The aim of this study was to assess the prevalence of ESGD and EGGD in a population of intact male horses in Italy, and to establish presence of correlation with age, breed, breeding and/or sports activity, and the presence of clinical signs.

## 2. Materials and Methods

### 2.1. Horses

A cohort of 101 intact male horses with and without overt clinical signs of gastric ulcers were enrolled in the study over a two-year period (2019–2020). None of the horses were undergoing heavy training at the time of the initial endoscopy. Information on age, breed, breeding, physical activity, and the presence/absence of clinical signs usually associated with gastric ulcers (weight loss, colic, exercise intolerance, girthiness, and ill thrift [1,2,4]) were recorded for each horse: according to the owners, symptoms were present in 15/101 (15%). The animals were categorized retrospectively into age groups: “young”, aged between 4 and 10 (47/101, 47%); “adult”, aged between 11 and 20 (43/101, 43%); “old”, aged 21 years and above (11/101, 10%). Gastroscopies were performed at the request of referring veterinarians or owners/trainers, as part of an assessment prior to the onset of the exercise or breeding season. All horses were exposed to some of the risk factors commonly associated with gastric ulcers (exercise, even if only low intensity; recent move from one stable to another; stress; etc.) [1,2,3,4,5,7,13]. Before performing the examination, an oral informed consent was obtained from the owner or the rider. The gastroscopy was executed before the beginning of the breeding season, and all breeding stallions had been used as sires for artificial insemination (AI), natural servicing, or both for at least one year before the inclusion. At the time of the gastroscopy, the horses were starting to be used again for semen collection once or twice per week, to prepare them for the upcoming breeding season. Some horses were used for show jumping (16/101), others for endurance (2/101), trekking (7/101), reining and similar western riding exercises (13/101), working equitation (2/101), and horse shows (5/101). It was the beginning of the sport season, and exercise intensity was similar: none of the animals underwent heavy training and long-distance traveling. They were exercised under the saddle once or twice per week, for less than one hour each day, and lunged or trained with a walker, on the other days, for less than twenty minutes each day. Horses used for both exercise and breeding underwent comparable workloads, with semen collection scheduled based on the demands of their training, but never less than once per week. Diet and stabling of the horses did not change during the year, with minimal adjustments made based on the demands on the breeding and performance season. All males were housed in boxes, with periodic access to the paddock (two or three times per week, for a few hours during the day), depending on their behavior. They were fed hay two to three times a day, and a commercial balanced feed twice a day.

Gastroscopy was conducted following established protocols [1,2,18,19], using a portable processor (Tele Vet X Led, Karl Storz, Tuttingen, Germany) and a 3 m long scope (60130PKS, Karl Storz, Tuttingen, Germany). Videos of the examination were recorded for subsequent assessment and archiving. Horses were fasted for at least 16 h and water was withheld for at least 4 h before examination [1,2,18,19]. After sedation with xylazine (0.25–1.1 mg/kg IV, Nerfasin, P.H. Farmaceutici, Milan, Italy) and using a twitch, the scope was introduced in the ventral nasal meatus and passed through the pharynx and the esophagus up to the stomach. After distending the stomach with air, the mucosa was cleaned with water which was passed through the working channel of the endoscope to remove any feed material. The gastroscopy was recorded, and all videos were coded using a numeric system and sent for evaluation, at the end of the study, to an investigator blinded to the name and the breeding and exercise history of the horse. The presence and severity of ESGD was recorded using the scoring system proposed in the ECEIM Consensus Statement [2]. Due to the current lack of a scoring system for EGGD, this condition was recorded only as present/absent, with a macroscopic description of the lesions and their localization [1,2]. Horses were considered positive for EGGD with any kind of alteration of the glandular mucosa (hyperemia, erosions, ulcers). ESGD was considered positive for grades of at least 2/4 [20].

### 2.2. Statistical Analysis

Normality of age distribution and grading of ESGD was tested using Shapiro–Wilk test. Simple logistic regression was used to determine the effect of age groups, breed, breeding and exercise history, and clinical signs on the presence of ESGD or EGGD. Multiple logistic regression was used to determine the effect of the combination of age and breed and breeding and exercise history on the same parameters. Differences in presence and severity of ESGD and EGGD between groups (horses used for breeding or not, for sports or not and animals with and without clinical signs) were determined using Wilcoxon rank sum test. Statistical significance was set at *p* < 0.05. Statistical analysis was performed using RStudio 2023.12.1 [21].

## 3. Results

The 101 male horses included in the study were aged between 4 and 28 years of age (median 10; interquartile range: 6–15). Considering the breeding career, 48/101 (48%) were used as sires, while 53/101 (52%) were not, either because they were not yet approved by their studbook or deemed uninteresting from a reproductive perspective. A total of 56/101 (55%) horses were not used for exercise, while 45/101 (45%) were involved in physical activity, predominantly show jumping (16/101, 16%), followed by reining and other western riding disciplines (13/101, 13%), trekking (7/101, 7%), shows (5/101, 4%), working equitation (2/101, 2%), and endurance (2/101, 2%). A total of 11/101 (11%) were used for both activities at the same time (above all, show jumping horses), while 19/101 (19%) were not used for either activity, mostly retired horses that were never approved by their studbooks. Distribution based on breed and age groups is reported in Table 1, and on breeding and exercise activity in Table 2.

Clinical signs compatible with gastric ulcers (exercise intolerance, poor body condition score, recurrent colic) were reported by the owners in 15/101 (15%) horses. Management in these were similar to the others: they were stabled in box, fed hay two or three times per day, and fed a commercial balanced supplementary feed twice. Four of them were young, nine adult, and two old; four were Arabians, three Italian saddlebred, two Murgese and the others were Dutch saddlebred, German saddlebred, Haflinger, Lusitano, Maremmano, and mixed breed. Eleven were not used for reproduction, eight were sport horses, and two were employed in both activities at the same time. ESGD was present in 13 of these animals: 2 were diagnosed with grade 2, 1 with grade 3, and 10 with grade 4. EGGD was detected in six of them.

**Figure 1:**
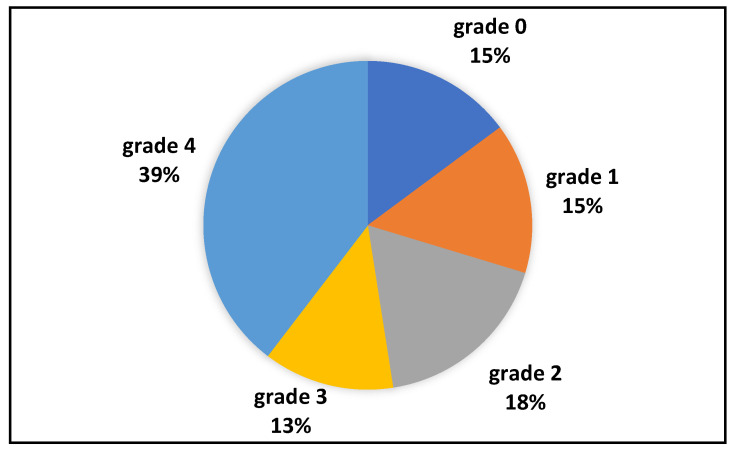
Prevalence of ESGD in the population; grading was performed according to the literature [1,2]: grade 0: normal mucosa; grade 1: hyperkeratosis; grade 2: small lesions, single or multiple; grade 3: large lesions, single or multiple; grade 4: areas of deep ulceration.

ESGD (see Figure 1) was present in 71/101 (70%), with grade 2 in 18/101 (17%), grade 3 in 13/101 (13%), and grade 4 in 40/101 (40%). Of the 30/101 (30%) horses negative for ESGD, 15/101 (15%) were grade 0, and 15/134 (15%) grade 1. EGGD was recorded, as only flat areas of hyperemia of the glandular mucosa, in 36/101 (36%) horses: 33 of these were also positive for ESGD. The distribution of the severity of ESGD and presence of EGGD in horses used for breeding, exercise, or both can be found in Table 3.

None of the parameters evaluated were normally distributed. Statistically significant differences were found for the presence of EGGD between horses that performed any kind of physical activity and those that did not (*p* = 0.04), between animals used for both exercise and breeding and the others (*p* = 0.04), and between the same group and those used only for breeding (*p* = 0.03). The severity of ESGD was statistically different between horses with and without clinical signs (*p* = 0.03), with higher grades found in the first group. Simple and multiple logistic models show no statistically significant effect of any of the parameters evaluated (breed, age, clinical signs, breeding, and exercise activity alone or in combination) on the presence of ESGD, while the same models show that exercise influences positivity for EGGD alone (odds ratio: 2.39; confidence interval: 1.05–5.58; *p* = 0.04) or when combined with breeding (breeding: odds ratio 1.55, confidence interval 0.61–4.13, *p* = 0.4; exercise: odds ratio 2.90, confidence interval 1.15–7.74, *p* = 0.027).

## 4. Discussion

Equine Gastric Ulcer Syndrome (EGUS) is a worldwide disease that affects all categories of horses, with varying prevalence rates. Exposure to stressful activities or management systems is believed to be one of the potential causes [1,2,3,13]. Breeding activity, especially the associated management is a stressful situation for horses [15,22]; however, during the breeding season, semen collection does not have a long-lasting effect on a horse’s stress levels [23]. Other studies have detected higher concentrations of cortisol in breeding stallion, with changes especially around the time of ejaculation [23,24]. Intact males are generally more nervous and excitable than geldings and mares, especially in the presence of other horses.

The aim of this study was to evaluate the prevalence of ESGD and EGGD in males and establish whether their breeding or exercise career may promote the development of gastric diseases.

Most of the horses in this population were non-breeding males (53/101), and enrolled in the group defined as “young” (4–10 years, 47/101). This reflects the normal situation, where younger horses are not gelded, when they have good pedigree, and their sport potential is still unknown. When they get older, owners may decide to geld them, due to behavioral or health concerns, especially when not approved as breeding stallions by their stud book [25]. 

The prevalence of ESGD is quite high in this population, similarly to other reports on sport horses [1,2,4], but higher than in pleasure horses, and those generally considered at decreased risk [2,16,26,27]. The occurrence of EGGD in this cohort, on the other hand, was lower than what has been reported by others [1,2,14,27]. A similar overall prevalence of EGUS was found in a study on Thoroughbred breeding mares at pasture, where 70.2% of animals were affected by gastric ulcers. The high prevalence of gastric ulceration in mares and in this study population highlights the need to further define the possible relationship between gastric ulcerations and breeding management. 

The procedures normally used in horse breeding programs can have a negative impact on the stress levels of animals [15,22]. In stallions, such stress seems to be transient [28], but results are variable, with other studies finding increased levels of cortisol in breeding stallions compared to mares and geldings [15,22,24,28]. 

To the best of our knowledge, an association between testosterone levels and gastric ulcers has never been investigated; however, one study found a negative correlation between hair cortisol and ESGD, possibly indicating a negative feedback mechanism secondary to high blood cortisol that reduces the synthesis of this hormone in the hair shaft [29]. These results could explain the findings of high prevalence of ESGD in our population [24].

In this cohort, breeding history on its own did not emerge as a determining factor either for ESGD or EGGD. Only exercise, alone or in combination with breeding, influenced the presence of EGGD. These findings are in accordance with the current literature: EGGD is affected by the number of days per week horses were trained and also by the competition season [1,2,3,13,14,30]. Despite variations in activity type in our cohort, the intensity of the exercise was similar, with horses trained for fewer than five hours per week, mainly once a day for one hour or less. Given that the number of horses performing each activity was too low, and the intensity was similar across the population, it was not possible to conduct a statistical analysis correlating the type of activity or its intensity to the presence of gastric ulcers. Only 11/101 (11%) horses were used for both exercise and breeding. This reflects current trends, where males are used for breeding mainly at the end of their sport career [15]. Contrary to current findings [15,31], combining exercise and breeding increases the risk of developing EGGD in this population. Although the literature diverges regarding the influence of exercise on cortisol in stallions and its impact on the release of testosterone, exercise intensity seems to be the primary determinant of the levels of the first hormone [32]. In fact, the release of cortisol seems to be correlated with the presence of EGGD, leading to a greater sensitivity of the adrenocortical axis, as demonstrated by an increased cortisol response to ACTH stimulation in animals with EGGD. However, these studies investigated females and geldings, so a complete comparison with this population is not possible [33,34]. The level of exercise in this cohort is below the current reports found in the literature as risk factors for ESGD and EGGD, and combining exercise and breeding activity seems to have an effect only on EGGD, probably because of increased levels of stress. It needs to be considered, though, that all the horses used for both exercise and breeding were used to this type of regimen, and the current literature indicates that high-performing animals may be better adapted to their environment and therefore their stress response is probably weaker than that of other horses [35].

In our cohort, the management system used was not considered, but all horses had some form of either paddock turnout or exercise on a walker. The choice depended on how stallions behaved in the paddock: nervous animals were exercised daily on a walker, and they did not receive any paddock turnout. Gastric ulcers, though, appear to be correlated with gastric compression following higher intra-abdominal pressure during heavy exercise [3,30]. Paddock turnout and exercise on a walker are not considered risk factors for gastric ulcers, but stall confinement has been associated with a higher prevalence of gastric ulcers [1,2].

The low number of horses with clinical signs of gastric ulcers (15/101, 15%) is in line with the current literature findings [1,2,12,13,26]. In this population, the clinical signs described by the owner were recurrent colic, weight loss, exercise intolerance and girthiness, and ill thrift, and are related to the severity of ESGD. The high number of horses with disease but not presenting symptoms supports gastroscopy as being the gold standard for diagnosis [1,2,26]. Most clinical signs of gastric ulcers are subtle, and especially in horses not performing at high levels, exercise intolerance, and rideability issues may have been overlooked by owners and riders [1,2,36,37]. There are currently no studies evaluating the effect of gastric ulcers on the reproductive performance of stallions, but in this population, none of the owners of the breeding horses reported reduced fertility and the reproductive efficiency was not evaluated.

One limitation of this study stems from the different premises and management practices used. Although most of the horses were kept in individual boxes, and fed with hay at least twice daily, some of the younger horses were kept in paddocks, but isolated from the other horses. In any case, age does not influence either ESGD or EGGD, and few of these horses had a different management system.

Finally, performing the gastroscopy at the beginning of the breeding season may be considered a limitation of this study. This population, though, had already been used for semen collection or natural service two times a week at the moment of the examination, so that an effect of some form of breeding management could still be determined, if present. Further studies could be performed, evaluating the horses before, during, and at the end of the breeding season, to determine if different moments and intensities of the management could influence gastric ulcers in breeding stallions.

## 5. Conclusions

In this population of intact males, the prevalence of ESGD was higher than usually found in the literature for low-risk horses, but similar to what has been described for Thoroughbred breeding mares at pasture [17]. In contrast, EGGD was lower than in other reports [1,2,4]. Breeding history did not influence the presence of gastric ulcers in either mucosa, while exercise only impacted on the likelihood of EGGD, similarly to when combining breeding and exercise. There were clinical signs only in a small number of horses, and such signs are indicative of severity of ESGD, confirming the need for a gastroscopic examination to diagnose the presence of gastric ulcers [1,2]. None of the owners reported reduced fertility in this population, but no data were available on the breeding soundness examination in the breeding stallions, and there are currently no objective assessments of the reproductive performance of horses with and without gastric ulcers. These results thus highlight the need for further studies on the influence of breeding activity on the development of gastric ulcers in horses, and also on the possible effects of gastric health on the fertility of mares and stallions.

## Figures and Tables

**Table 1:** Distribution of the population according to breed and age.

Breed	Number	Percentage
AngloArabian	1	1%
Friesian	3	3%
Haflinger	1	1%
Lusitano	1	1%
Maremmano	1	1%
Murgese	2	2%
Paint	2	2%
Pony	1	1%
Spanish	4	4%
Arabian	27	27%
Quarter Horse	12	12%
Mixed breed	2	2%
Belgian Warmblood	3	3%
French Warmblood	1	1%
Italian Saddlebred	19	18%
KWPN	2	2%
German Warmblood	8	8%
Italian Heavy horse	3	3%
Standardbred	8	8%
Total	101	100%
**Age group**	**Number**	**Percentage**
Young	47	47%
Adult	43	43%
Old	11	10%
Total	101	100%

**Table 2:** Distribution of the population according to breeding, exercise activity and age groups.

Category	Number (Percentage)
Not used for breeding	53 (52%)
Not used for exercise	19 (18%)
Young	14 (14%)
Adult	4 (4%)
Old	1 (1%)
Used for exercise	34 (34%)
Young	16 (16%)
Adult	16 (16%)
Old	2 (2%)
Used for breeding	48 (48%)
Not used for exercise	37 (37%)
Young	13 (13%)
Adult	17 (17%)
Old	7 (7%)
Used for exercise	11 (11%)
Young	4 (4%)
Adult	6 (6%)
Old	4 (4%)
Total	101 (100%)

**Table 3:** Distribution of the severity of ESGD and presence of EGGD in horses used for breeding, exercise, or both.

Category	ESGD Grade 0	ESGD Grade 1	ESGD Grade 2	ESGD Grade 3	ESGD Grade 4	EGGD Positive	EGGD Negative
Breeding							
Yes	9/101 (9%)	4/101 (4%)	7/101 (7%)	6/101 (6%)	21/101 (21%)	17/101 (17%)	31/101 (31%)
No	6/101 (6%)	11/101 (11%)	11/101 (11%)	7/101 (7%)	19/101 (18%)	19/101 (18%)	34/101 (34%)
Exercise							
Yes	8/101 (7%)	4/101 (4%)	6/101 (6%)	5/101 (5%)	22/101 (22%)	24/101 (24%)	21/101 (21%)
No	7/101 (7%)	11/101 (11%)	12/101 (12%)	6/101 (6%)	18/101 (18%)	41/101 (41%)	15/101 (15%)
Both							
Yes	3/101 (3%)	0/101 (0%)	0/101 (0%)	1/101 (1%)	7/101 (7%)	4/101 (4%)	7/101 (7%)
No	12/101 (12%)	15/101 (15%)	18/101 (17%)	12/101 (12%)	33/101 (33%)	61/101 (60%)	29/101 (29%)

## Data Availability

Data available on request from the authors.
